# Complete Genome Sequence of *Lactobacillus oris* J-1, a Potential Probiotic Isolated from the Human Oral Microbiome

**DOI:** 10.1128/genomeA.00970-16

**Published:** 2016-09-15

**Authors:** Fang Jia

**Affiliations:** Department of Stomatology, Xintai People Hospital, Xintai, People’s Republic of China

## Abstract

Lactobacilli can exert health-promoting effects in the human oral microbiome through many mechanisms, including pathogen inhibition, maintenance of microbial balance, immunomodulation, and enhancement of the epithelial barrier function. Here, we present the complete genome sequence of a potential probiotic, *Lactobacillus oris* J-1, that was isolated from the oral cavity of a health child.

## GENOME ANNOUNCEMENT

Lactobacilli, the acidophilic and aciduric Gram-positive bacteria of the genus *Lactobacillus*, belong to the indigenous microflora of humans and colonize various parts of the human body (1). They are known to play an important role in the maintenance of human health by stimulating natural immunity and contributing to the balance of microflora, mainly through competitive exclusion and antimicrobial activity against pathogenic bacteria (2). *Lactobacillus oris* J-1, which was isolated from the oral cavity of a healthy child, was found to be beneficial as a potential probiotic. Here, we present the complete sequence of this strain, which is also the first complete genome sequence of *L. oris*.

Genomic DNA from *L. oris* J-1 was extracted using the Wizard Genomic DNA purification kit (Promega, USA). The quantity and quality of genomic DNA were evaluated on a Qubit fluorometer (Thermo Fisher, USA). A 10 kb insert SMRT-bell library was constructed and then sequenced using a Pacific Biosciences (PacBio) RS II sequencer (Pacific Biosciences, CA). All of the filtered sequences were *de novo* assembled using SMRT analysis software version 2.3.0 (Pacific Biosciences), and it resulted in one circularized complete chromosome sequence and two more plasmids, with more than 270-fold coverage. Putative protein-coding sequences were predicted using Glimmer 3.0 (3). Gene functional annotation was performed using BLASTp with KEGG, COG, Swiss-Port, TrEMBL, nr, and GO databases. tRNA-encoding genes and rRNA operons were found by using tRNAscan (4) and RNAmmer softwares (5). Noncoding RNAs (ncRNAs) were predicted using the NCBI Prokaryotic Genomes Annotation Pipeline (PGAP).

The 3,236,616 bp genome is composed of one circular chromosome and two plasmids with a G+C content of 51.3%, 49.5%, and 48.8%, respectively. The coding regions cover 83.7% of the genome, including 3,133 protein coding genes, 95 tRNAs, and seven rRNA operons. Functional annotation of the genome revealed the presence of genes responsible for the two major pathways of lactic acid production, the Embden-Meyerhof-Parnas pathway and phosphoketolase/pentose phosphate pathway (6), which could utilize hexose and pentose as carbon sources simultaneously. We also identified all the necessary upstream genes for xylose utilization, including the xylose utilization pathway (*xyl*AB) and xylose transport system (*xyl*EGTH). Genome mining analysis using antiSMASH 3.0 (7) and dbCAN (8) revealed the presence of seven putative gene clusters responsible for the production of diverse secondary metabolites in the complete genome sequence of J-1. Of these, putative microcin synthesis is highly conserved in other lactic acid strains.

### Accession number(s).

The complete genome sequence of *Lactobacillus oris* J-1 has been deposited at GenBank under accession no. CP014787, CP014788, and CP014789. The strain J-1 has been deposited at the China Center for Type Culture Collection under the accession no. CCTCC AB 2016096.
